# An Important Function of Petrosiol E in Inducing the Differentiation of Neuronal Progenitors and in Protecting Them against Oxidative Stress

**DOI:** 10.1002/advs.201700089

**Published:** 2017-07-05

**Authors:** Jing Liu, Linlin Wang, Yuguo Du, Sijin Liu

**Affiliations:** ^1^ State Key Laboratory of Environmental Chemistry and Ecotoxicology Research Center for Eco‐Environmental Sciences Chinese Academy of Sciences Beijing 100085 P. R. China; ^2^ University of Chinese Academy of Sciences Beijing 100049 P. R. China

**Keywords:** Akt, arsenic, Erk1/2, neurite outgrowth, Nrf2, Petrosiol E

## Abstract

Insufficient endogenous neurotrophin supply contributes to neurodegeneration. Meanwhile, neuronal injuries are also attributed to oxidative stress upon toxin exposure. Thus, reconstruction neurite extension and antioxidative stress are the potential strategies for ameliorating neuronal injuries. However, there is no well‐defined therapeutic developed in this regard. In search of such therapeutics, Petrosiol E is identified here as a potent inducer to guide the differentiation of neuronal progenitor cells. Petrosiol E also considerably promotes embryonic stem cell differentiation into neural ectoderm features. Moreover, Petrosiol E reveals an antioxidant function to protect cells from oxidative stress induced by arsenic. Moreover, the molecular mechanism underlying Petrosiol E‐induced neuronal differentiation is uncovered: (a) enhancement of NF‐E2‐related factor 2 (Nrf 2) activity in driving neuronal differentiation; (b) diminishment of oxidative stress. Petrosiol E activates the mitogen‐activated protein kinase and serine/threonine kinase signaling to enhance the activity of Nrf 2. As a result of enhanced Nrf 2 activity, neuronal differentiation is accelerated, and the cellular antioxidation responses are also enforced, even under arsenic‐induced neurotoxicity. Together, the combined results unveil a desirable role of Petrosiol E in driving neuronal differentiation and in combating oxidative stress. This study would open an avenue to develop new therapeutics based on Petrosiol compounds to treat neurodegenerative diseases.

## 1 Introduction

Neuronal dysfunction of synaptic transmission and dendritic degeneration represent common causes for neurodegenerative diseases (NDs). NDs, e.g., Alzheimer's disease (AD) and Parkinson's disease (PD), are the prevalent dementing disorders, causing considerable morbidity and mortality in the world.^1^ For example, more than 47 million people are suffering from AD worldwide, and the number is expected to reach 131 million by 2050.^2^ Neuron loss and oxidative stress are considered as the common causes for the development of NDs.^3^ On one hand, neuron loss results in cognitive deterioration and memory loss in AD.^4^ On the other hand, amyloid‐β (Aβ) peptide aggregation, deriving from amyloid precursor protein (APP), remarkably accelerates the development and progression of AD.^5^, ^6^ Moreover, Aβ peptide aggregation gives rise to significant oxidative stress, leading to Aβ neurotoxicity, associated with neuronal death through depleting intracellular glutathione.^7^

Therefore, to treat AD or ameliorate its symptoms and complications, several types of agents are being developed thus far. Among these, nerve growth factor (NGF) has been proved as effective regent to promote neuron plasticity; however, low penetration across the brain blood barrier (BBB) limits its practical applications.^8^ Although antibodies (Abs) targeting aspartyl protease β‐site APP cleaving enzyme (BACE_1_)^9^ and Aβ (named crenezumab)^10^ that reduce Aβ accumulation have been developed, roughly 0.1–0.2% uptake in the brain largely restricts their widespread applications.^11^ Several antioxidants, e.g., polyphenols and flavonoids, were examined to eliminate oxidative damages.^12^, ^13^ Nonetheless, no effective drugs for AD have been developed from the perspective of combating oxidative stress for nearly two decades.^14^ Thereby, new therapeutics for AD is urgently needed.

Petrosiol A–E, natural products extracted from the Okinawan Sponge *Petrosia strongylata*, harbor similar unusual diyne tetraol skeleton with different side‐chain lengths.^15^ Preliminary bioactivity screening of Petrosiol compounds exhibited a possible ability to promote neuron differentiation.^15^ The small quantity of these products in natural resources greatly restrains the pharmaceutical exploration and their potential translation into clinical applications as well. The unique structures of these compounds bring about substantial difficulties in synthesizing the artificial ones. Given their great value in pharmaceutics in translational medicine, several groups have been exerting great efforts to artificially synthesize these active natural products. We recently achieved the first total synthesis of (‐)‐Petrosiol E in ten steps starting from the chiral template D‐xylose with an overall yield of 32%.^16^ This efficient synthesis of Petrosiol E offers an opportunity to facilitate the mechanistic studies on its role in guiding neuron differentiation. Thus far, several cell lines have been established for in vitro studies in neuroscience, including PC12 cells.^17^, ^18^ PC12 cells are recognized as a cell model representative of neuronal progenitor cells.^17^, ^18^ Therefore, PC12 cells could be induced to differentiate into neuronal cells, and have been wildly used to investigate basic neurobiology and to evaluate chemical‐mediated effects on neurite outgrowth.^19^, ^20^, ^21^ To this end, in this study, we tested the functions of Petrosiol E in proneuron differentiation and antioxidative stress in PC12 cells. We identified a dual role of Petrosiol E in potentiating the differentiation of neuronal progenitors and in protecting them from oxidative stress. This study may prove to be a promising strategy through developing Petrosiol compounds to treat NDs.

## 2 Results

### 2.1 Screening of Concentrations of Petrosiol E to Induce PC12 Cells

**Figure 1:**
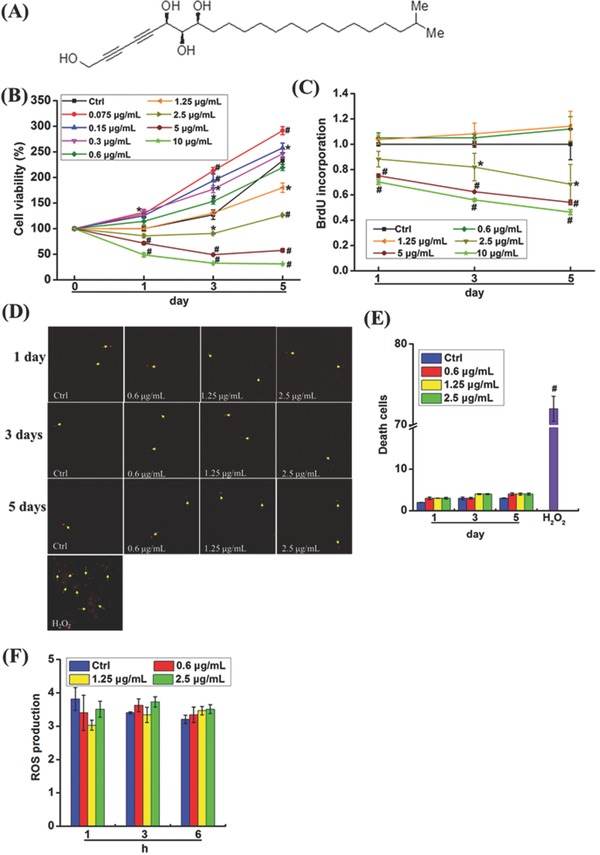
Screening of sublethal concentrations of Petrosiol E in PC12 cells. A) The chemical structure of Petrosiol E. B) Cell viability was determined through the MTT assay in PC12 cells treated with Petrosiol E at different concentrations for 1, 3, and 5 d (*n* = 6). C) Cell proliferation was assessed by the BrdU incorporation assay at indicated concentrations for 1, 3, and 5 d (*n* = 6). D) Dead cells were determined by PI staining after Petrosiol E treatment at 0.6, 1.25, and 2.5 µg mL^−1^ for 1, 3, and 5 d (*n* = 6). H_2_O_2_ (at 5 mmol L^−1^) was used as a positive control to induce cell death. E) Quantitative analysis of dead cells after PI staining (*n* = 6). F) Levels of ROS were detected via DCFH‐DA probe in PC12 cell upon Petrosiol E exposure (*n* = 6). Experiments were repeated for three times. *: *P* < 0.05, relative to untreated control. #: *P* < 0.001, relative to untreated control.

To evaluate the function of Petrosiol E in inducing neuronal progenitor cell differentiation, PC12 neuronal progenitor cells were used as the model to test this. To find out the feasible concentrations of Petrosiol E in cellular experiments, we first measured the cell viability of PC 12 cells through the 3‐(4,5‐dimethyl‐2‐thiazolyl)‐2,5‐diphenyl‐2‐H‐tetrazolium bromide (MTT) assay. Overall, Petrosiol E manifested a mild effect on cell viability over the time course at the concentrations below 5 µg mL^−1^, and a slight increase of cell viability was observed at low concentrations, such as 0.075 and 0.15 µg mL^−1^ (**Figure** **1**B). Meanwhile, an inhibition of cell viability was found with concentrations at 5 and 10 µg mL^−1^ (Figure 1B, *P* < 0.05). For example, Petrosiol E inhibited PC12 cell viability by ≈25% (*P* < 0.001), and further repressed cell viability by 50% (*P* < 0.001) at 10 µg mL^−1^ after 1 d treatment (Figure 1B, *P* < 0.001). The inhibition on cell viability was enhanced at 5 and 10 µg mL^−1^ over the time course to day 5 (Figure 1B, *P* < 0.001). To understand the changes of cell viability upon Petrosiol E, cell division and cell death were determined. First, BrdU incorporation assay was used to assess cell proliferation. Consistent with the MTT results (Figure 1B), Petrosiol E at 0.6 and 1.25 µg mL^−1^ revealed little impact on cell growth, and 2.5 µg mL^−1^ Petrosiol E mildly suppressed cell proliferation over the time course, especially at day 3 and day 5 (Figure 1C, *P* < 0.05). However, much greater suppression on cell proliferation was observed at 5 and 10 µg mL^−1^ (Figure 1C, *P* < 0.001). Afterward, to figure the reason for inhibition on cell viability and proliferation by Petrosiol E at higher concentrations, we further looked into the occurrence of cell death by propidium dodide (PI) staining. As shown in Figure 1D,E, no significant cell death was observed in PC12 cells treated with Petrosiol E at 0.6, 1.25, and 2.5 µg mL^−1^ (*P* > 0.05), suggesting no toxicity of Petrosiol E to PC12 cells at these concentrations. Nevertheless, increased cell death was detected at 5 and 10 µg mL^−1^ after 24 h (Figure S1A,B in the Supporting Information, *P* < 0.001). Here, we used H_2_O_2_ as a positive control to induce cell death (Figure 1D,E, *P* < 0.001). In support of this finding, no significant burst of reactive oxygen species (ROS) generation was found in cells upon Petrosiol E treatment at 0.6, 1.25, and 2.5 µg mL^−1^ at different time points (Figure 1F, *P* > 0.05). Collectively, Petrosiol E did not elicit toxicity to PC12 cells at 2.5 µg mL^−1^ and lower than this concentrations, implying a potential role of Petrosiol E in inducing neuronal progenitor differentiation by means of repressing their proliferation. In the meantime, Petrosiol E could cause slight toxicity to cells at high concentrations greater than 5 µg mL^−1^.

### 2.2 Petrosiol E Induces Neuronal Differentiation of PC12 Cells and Facilitates Neuron Ectoderm Differentiation of Embryonic Stem (ES) Cells

**Figure 2:**
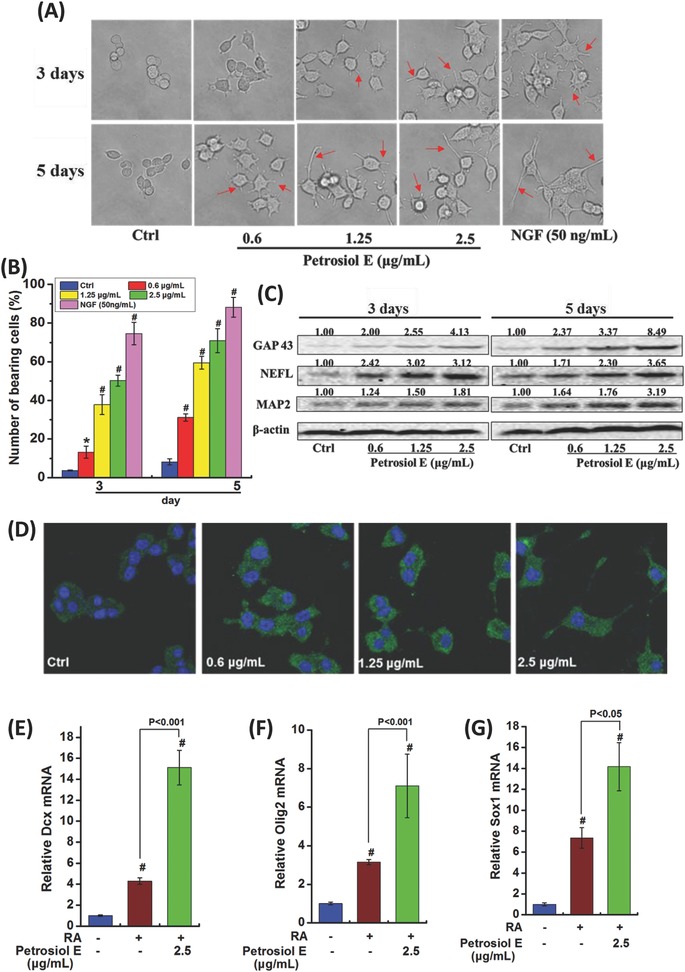
Petrosiol E promoted neurite outgrowth in PC12 cells and neural ectoderm differentiation in ES cells. A) The morphology changes of PC12 cells upon Petrosiol E induction at various concentration for 3 and 5 d. NGF (at 50 ng mL^−1^) was used as positive control to induce neurite outgrowth. B) Quantitative analysis of neurite bearing cells (*n* = 6 × 3). C) Western blot analysis of GAP 43, NEFL, and MAP2 in PC12 cells upon Petrosiol E at 0.6, 1.25, and 2.5 µg mL^−1^ for 3 and 5 d. D) Immunofluorescence of NEFL (shown in green) in PC12 cells upon Petrosiol E treatment at various concentrations for 3 d. The relative mRAN levels of E) Dcx, F) Olig2, and G) Sox1 in differentiated ES cells upon RA with or without 2.5 µg mL^−1^ Petrosiol E. All the experiments were performed for three times. *: *P* < 0.05, relative to control. #: *P* < 0.001, relative to control.

To address the above hypothesis, neuronal differentiation of PC12 cells was conducted. As shown in **Figure** **2**A,B, Petrosiol E treatment for 3 d greatly increased the number of neurites in cells (denoted by red arrows) at 0.6, 1.2, and 2.5 µg mL^−1^, especially at 2.5 µg mL^−1^ (*P* < 0.001). A greater phenotype of neurite outgrowth was observed after 5 d treatment with Petrosiol E (Figure 2A,B, *P* < 0.001). Specifically, ≈50% of cells showed neurite outgrowth upon Petrosiol E at 2.5 µg mL^−1^ on day 3, and 70% of cells manifested such a phenotype on day 5 (Figure 2A,B, *P* < 0.001). Moreover, a clear dose dependency was demonstrated from 0.6 to 1.2, and 2.5 µg mL^−1^ (Figure 2A,B, *P* < 0.05). NGF at 50 ng mL^−1^ was used here as the positive control to induce neuronal differentiation (Figure 2A,B, *P* < 0.001). These results therefore indicated that Petrosiol E harbored a robust ability to promote neuronal differentiation.

To validate this finding on Petrosiol E's ability to enhance neurite outgrowth, more neuronal differentiation markers including growth associated protein 43 (GAP 43), neurofilament light (NEFL), and microtubule associated protein 2 (MAP2) were analyzed after Petrosiol E treatment through Western blotting. GAP 43 plays a key role in regulating the growth of axon and the formation of cell connection.^22^ NEFL is an important component of neuron, necessary for the assembly and maintenance of the axonal cytoskeleton.^23^ MAP2, another specific surrogate for neuronal differentiation, is involved in the maintenance of neuronal polarity.^24^ In agreement with the results of neurite outgrowth (Figure 2A,B), Petrosiol E greatly enhanced the concentrations of GAP 43, NEFL, and MAP2 after 3 and 5 d treatment (Figure 2C). To substantiate the Western blotting results, immunofluorescence staining of NEFL was performed. As shown in Figure 2D, immunofluorescence staining results manifested an increased NEFL level (shown in green) in PC12 cells after Petrosiol E treatment at 0.6, 1.2, and 2.5 µg mL^−1^. Thus, our data indicated that Petrosiol E revealed a great ability to promote neuronal differentiation.

Moreover, we elaborated the capability of Petrosiol E along with retinoic acid (RA) to induce neuronal differentiation of CCE ES cells. As shown in Figure 2E–G, Petrosiol E significantly accelerated RA‐induced neuron‐like differentiation, as evidenced by the greater induction of neural ectoderm markers, including doublecortin (Dcx), oligodendrocyte transcription factor 2 (Olig2), and sex‐determining region Y (SRY)‐box (Sox1), compared to RA treatment alone (*P* < 0.05). These results would therefore define an important function of Petrosiol E in promoting ES cell differentiation into neural ectoderm, analogous to the results observed in PC12 cells.

### 2.3 NF‐E2‐Related Factor 2 (Nrf2) Is Necessary for Petrosiol E‐Induced Neuron‐Like Differentiation

**Figure 3:**
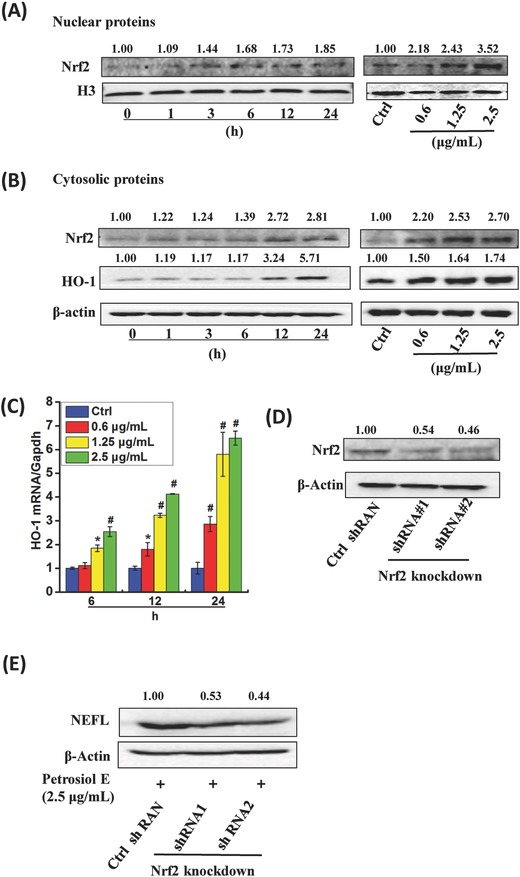
Alterations of Nrf2 concentration in PC12 cells upon Petrosiol E. A) Left panel, the concentration of nuclear Nrf2 in PC12 cells treated with Petrosiol E at 2.5 µg mL^−1^ at different time points. Right panel, the concentration of nuclear Nrf2 in PC12 cells treated with Petrosiol E at 0.6, 1.25, and 2.5 µg mL^−1^ for 24 h. B) Left panel, the concentrations of cytoplasmic Nrf2 and HO‐1 in PC12 cells after Petrosiol E treatment at 2.5 µg mL^−1^ for different time. Right panel, the concentrations of cytoplasmic Nrf2 and HO‐1 in PC12 cells with Petrosiol E treatment at 0.6, 1.25, and 2.5 µg mL^−1^ for 24 h. C) The mRNA levels of HO‐1 expression upon various concentrations of Petrosiol E for 6, 12, and 24 h (*n* = 6). D) The Nrf2 mass in PC12 cells after Nrf2‐knockdown for 24 h. E) NEFL expression in Nrf2 knockdown cells upon 2.5 µg mL^−1^ Petrosiol E for 3 d. All the experiments were repeated for three times.*: *P* < 0.05, relative to control. #: *P* < 0.001, relative to control.

To validate the above findings, we further looked for the mechanisms underlying Petrosiol E‐induced neuronal differentiation. Nrf2 is a master transcription factor in protecting cells from oxidative stress by orchestrating the expression of oxidative stress‐related genes.^25^ Importantly, previous studies also demonstrated that Nrf2 is indispensable for neuronal differentiation.^26^ To this end, we investigated the likely contribution of Nrf2 to Petrosiol E‐induced neuronal differentiation. As shown in **Figure** **3**A, Petrosiol E (at 2.5 µg mL^−1^) greatly increased the concentration of Nrf2 in the nuclear portion over the time course from 1 to 24 h, relative to untreated cells. Moreover, a dose‐dependent increase of nuclear Nrf2 concentration was observed at 0.6, 1.25, and 2.5 µg mL^−1^ (Figure 3A). In analogy to the increase of nuclear Nrf2 level, the cytosolic Nrf2 level was also greatly enhanced by Petrosiol E over the time course and in a dose‐dependent manner (Figure 2B). In support of these data, a downstream target of Nrf2, heme oxygnase‐1 (HO‐1),^27^ was similarly evoked by Petrosiol E at the protein level and mRNA level as well (Figure 2B,C, *P* < 0.05). Similar to Nrf2, the increase of HO‐1 showed a clear time‐ and dose‐dependency (Figure 2B,C, *P* < 0.05). In an effort to substantiate the role of Nrf2 in promoting neuronal differentiation induced by Petrosiol E, endogenous Nrf2 expression was knocked down using the approach of shRNA. As shown in Figure 3D, the endogenous Nrf2 concentration was greatly diminished in two transfectants, namely, shRNA#1 and shRNA#2, compared to the transfectant with scrambled control shRNA. Subject to Nrf2 knockdown, the NEFL concentration was consequently reduced after Petrosiol E treatment at 2.5 µg mL^−1^ (Figure 3E), pinpointing a crucial role of Nrf2 in enhancing neuronal differentiation. These findings therefore indicated that Petrosiol E enhanced the level of Nrf2 to elicit neuronal differentiation.

### 2.4 Activation of Mitogen‐Activated Protein Kinase (Erk1/2) and Serine/Threonine Kinase (Akt) Drives Nrf2 for Neuron‐Like Differentiation under the Induction of Petrosiol E

**Figure 4:**
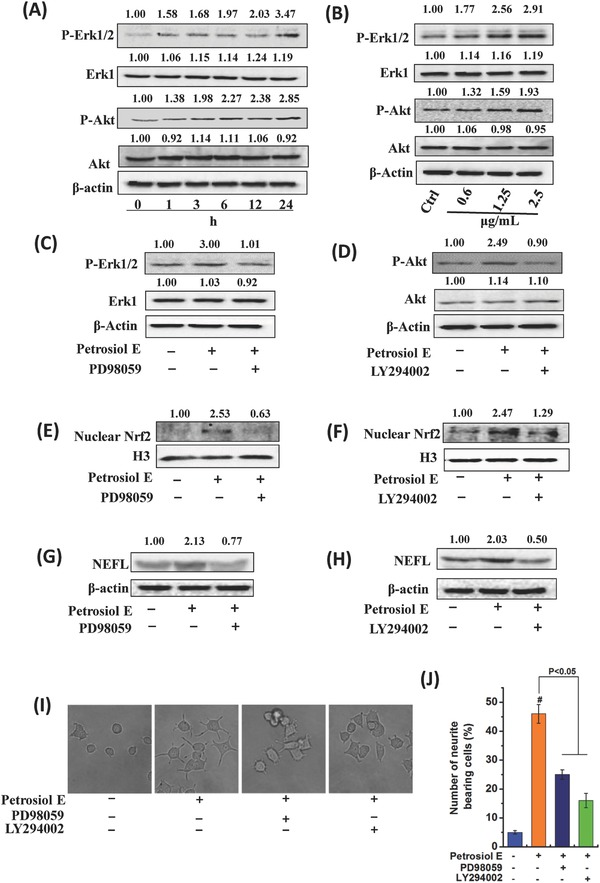
Activation of Erk1/2 and Akt in PC12 cells upon Petrosiol E. A) P‐Erk1/2 and P‐Akt levels in PC12 cells at different time points upon 2.5 µg mL^−1^ Petrosiol E. B) P‐Erk1/2 and P‐Akt mass in PC12 cells upon Petrosiol E at 0.6, 1.25, and 2.5 µg mL^−1^ for 24 h. C) P‐Erk1/2 concentrations in PC12 cells upon PD98059 at 1.5 µg mL^−1^ for 6 h. D) P‐Akt levels in PC12 cells treated with LY294002 (at 3 µg mL^−1^) for 6 h. Western blot analysis of nuclear Nrf2 (E) upon P‐Erk1/2 inhibitor PD98059 (at 1.5 µg mL^−1^) and F) P‐Akt inhibitor LY294002 (at 3 µg mL^−1^) pretreatment 1 h prior to Petrosiol E induction. The NEFL levels in PC12 cells upon G) PD98059 and H) LY294002 pretreatment 1 h prior to Petrosiol induction for 3 d. I) Cellular morphology of PC12 cells with PD98059 and LY294002 pretreatment prior to Petrosiol E induction. J) The number of neurite bearing cells with PD98059 and LY294002 pretreatment upon Petrosiol E induction. #: *P* < 0.001, relative to control.

Next, we endeavored to search for the upstream molecules that drive the increase of Nrf2. Although the upstream signaling to regulate Nrf2 has not been fully elucidated, a few studies suggested that Nrf2 is activated by Erk1/2 and Akt.^28^ We thus hypothesized that Erk1/2 and Akt could be involved in activating Nrf2 under the induction of Petrosiol E. To test this hypothesis, we determined their alterations in PC12 cells upon Petrosiol E. As shown in **Figure** **4**A, Petrosiol E enhanced the phosphorylation of Erk1/2 and Akt over the time course from 1 to 24 h. Meanwhile, a dose‐dependent increase of Erk1/2 and Akt phosphorylation was found in PC12 cells upon Petrosiol E at 0.6, 1.25, and 2.5 µg mL^−1^ (Figure 4B). These results pointed out the important contribution of Erk1/2 and Akt activation to Petrosiol E‐induced PC12 cell differentiation. To this end, we employed selective inhibitors for Erk1/2 and Akt to inhibit the kinase activities. Thereafter, kinase inhibition experiments were carried out. Analogous to our above assumption, Erk1/2 inhibitor PD98059 and Akt inhibitor LY294002 significantly reversed the increases of the phosphorylation of Erk1/2 and Akt induced by Petrosiol E, respectively (Figure 4C,D). As a consequence, the elevated concentration of nuclear Nrf2 was similarly reversed by Erk1/2 inhibitor PD98059 and Akt inhibitor LY294002, respectively (Figure 4E,F). These data therefore signified the regulation of Nrf2 by Erk1/2 and Akt under Petrosiol E‐induced neuronal differentiation.

To recognize the importance of this regulation in enhancing PC12 differentiation, we thereafter investigated the impact of Erk1/2 and Akt inhibition on cell differentiation. As shown in Figure 4G,H, Erk1/2 and Akt inhibition greatly reversed the induction of NEFL level by Petrosiol E. As a result, the neurite outgrowth was also compromised by more than 50% upon Erk1/2 and Akt inhibitors, compared to Petrosiol E (Figure 4I,J, *P* < 0.05). Therefore, our combined results demonstrated that Erk1/2 and Akt are necessary for Petrosiol E‐induced neuronal differentiation by enhancing the activity of Nrf2.

### 2.5 Protection of Petrosiol E for PC12 Cells against Arsenic‐Induced Oxidative Stress

Since Nrf2 is the master transcriptional factor to combat oxidative stress by driving a number of antioxidant genes in mammalian cells,^29^ we assumed the elevation of Nrf2 induced by Petrosiol E might also play a vital role in protecting PC12 cells against oxidative stress. To examine the assumption, arsenic was used as the inducer to elicit oxidative stress.^30^

**Figure 5:**
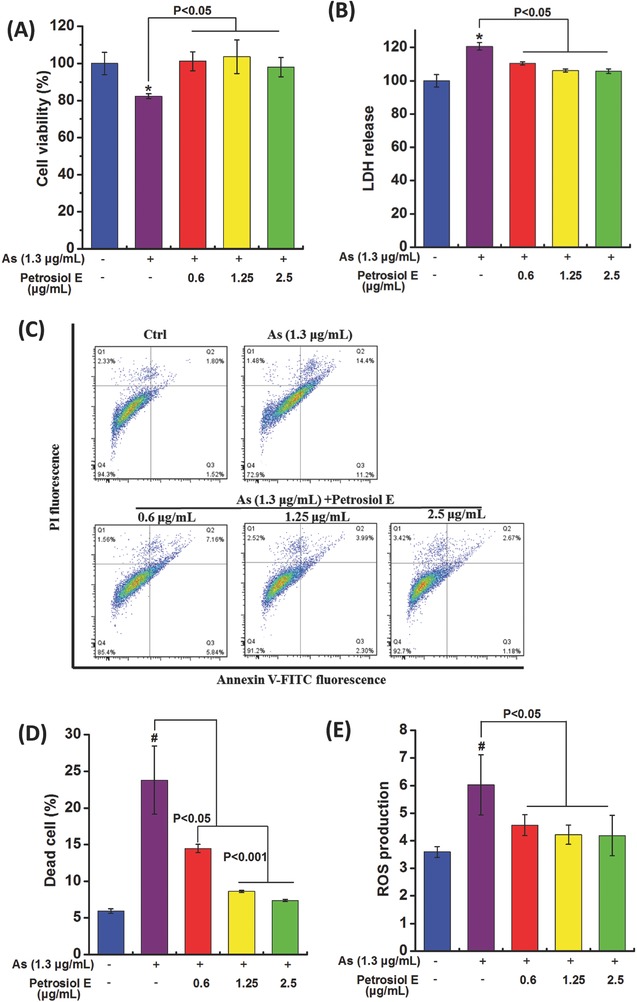
The protection of Petrosiol E from arsenic‐induced cytotoxicity. PC12 cells were pretreated with Petrosiol E at 0.6, 1.25, and 2.5 µg mL^−1^ for 1 h, and were further treated with arsenic at 1.3 µg mL^−1^ for additional 24 h. A) Cell viability, B) LDH release, C) cell death, D) quantitative analysis of dead cells (*n* = 6), and E) intracellular ROS levels were assessed (*n* = 6). All the experiments were performed for three times. *: *P* < 0.05, relative to control. #: *P* < 0.001, relative to control.

As shown in Figure S2 (Supporting Information), arsenic started to cause injuries to cell viability at 1.3 µg mL^−1^, with according impairment in the cellular morphology, in a dose‐dependent manner (*P* < 0.05). As exposure at 1.3 µg mL^−1^ induced a significant reduction of cell viability by ≈20% in PC12 cells, as reflected by the MTT assay (**Figure** **5**A, *P* < 0.05). However, the decrease of cell viability responding to arsenic treatment was significantly ameliorated with Petrosiol E pretreatment at 0.6, 1.25, and 2.5 µg mL^−1^, as evidenced by increased cell viability comparable to untreated cells (Figure 5A, *P* < 0.05). These results suggested a great role of Petrosiol E in antagonizing arsenic‐provoked toxicity to PC12 cells.

To substantiate the protective role of Petrosiol E, lactate dehydrogenase (LDH) release was determined as well. The LDH release was increased by 20% upon arsenic treatment at 1.3 µg mL^−1^ in PC12 cells, in comparison to untreated cells (Figure 5B, *P* < 0.05). Similar to the cytotoxicity results (Figure 5A), the LDH release was significantly repressed with Petrosiol E pretreatment nearly to the baseline level comparable to that in the untreated cells (Figure 5B, *P* < 0.05). In accordance with the above results, arsenic‐stimulated cell death was greatly restrained by almost 50% with Petrosiol E pretreatment at 0.6 µg mL^−1^ (Figure 5C,D, *P* < 0.05), and nearly to the baseline value with Petrosiol E pretreatment at 1.25 and 2.5 µg mL^−1^, as characterized by the flow cytometry (FACS) analysis using PI and Annexin V staining (Figure 5C,D, *P* < 0.001). Oxidative stress is the predominant mechanism underlying arsenic‐induced cytotoxicity.^30^ Consistent with previous reports, arsenic treatment significantly stirred the intracellular ROS production by 1.5‐fold, relative to untreated cells (Figure 5E, *P* < 0.001). Analogous to the cytotoxicity results, intracellular ROS generation was remarkably reversed with Petrosiol E pretreatment almost to the baseline level, compared to arsenic‐treated cells without preincubation of Petrosiol E (Figure 5E, *P* < 0.05). These results collectively uncovered a crucial function of Petrosiol E in combating arsenic‐conducted toxicity to PC12 cells.

### 2.6 Petrosiol E Protects PC12 Cells against Oxidative Stress by Elevating Cellular Antioxidant Activity

**Figure 6:**
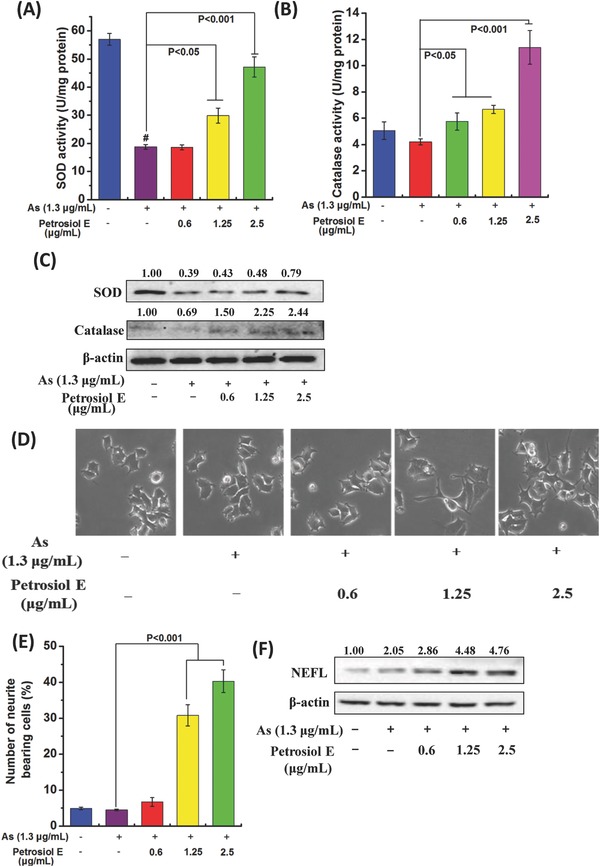
Petrosiol E protects cells against oxidative stress and promotes neurite outgrowth under arsenic exposure. PC12 cells were pretreated with Petrosiol E at 0.6, 1.25, and 2.5 µg mL^−1^ for 1 h, and were further treated with arsenic at 1.3 mg mL^−1^ for dictated time. Thereafter, A) SOD and B) catalase activities, and C) SOD and catalase concentrations were determined on day 1. Furthermore, D) cellular morphology, E) quantitative analysis of neurite bearing cells (*n* = 6), and F) NEFL levels were assessed on day 3. All the experiments were repeated for three times. #: *P* < 0.001, relative to control.

Afterward, we looked into the action of mode for Petrosiol E in protecting PC12 cells. Since superoxide dismutase (SOD) are the superoxide (O_2_^−^) scavenger and catalase are hydrogen peroxide (H_2_O_2_) scavenger,^31^ SOD and catalase activities were thereby determined. As shown in **Figure** **6**A, SOD activity was depleted by 70% in PC12 cells upon 1.3 µg mL^−1^ arsenic treatment, compared to untreated cells (*P* < 0.001). However, Petrosiol E pretreatment reversely enhanced the SOD activity by 1.5‐fold at 1.25 µg mL^−1^ (Figure 6A, *P* < 0.05) and by more than twofold at 2.5 µg mL^−1^, compared to arsenic‐treated cells without Petrosiol E pretreatment (Figure 6A, *P* < 0.001). Petrosiol E pretreatment at 0.6 µg mL^−1^ also showed little stimulating effect on SOD activity (Figure 6A). Compared to untreated cells (Figure 6B), 1.3 µg mL^−1^ arsenic mildly repressed the catalase activity. Nonetheless, Petrosiol E pretreatment indeed elevated the catalase activity in a dose‐dependent manner at 0.6, 1.25, and 2.5 µg mL^−1^, and the max value was observed at 2.5 µg mL^−1^ with nearly threefold induction, compared to arsenic‐treated cells without Petrosiol E pretreatment (Figure 6B, *P* < 0.05). The alterations of SOD and catalase were also confirmed at the protein levels, as characterized by Western blotting results (Figure 6C). In addition to the protection of cells from arsenic‐induced oxidative stress, Petrosiol E was also found to aid PC12 cell differentiation even under arsenic treatment. As shown in Figure 6D,E, the neurite outgrowth were observed in arsenic‐treated PC12 cells with the protection of Petrosiol E at 0.6, 1.25, and 2.5 µg mL^−1^, especially at 1.25 and 2.5 µg mL^−1^ (Figure 6D,E, *P* < 0.001). Moreover, NEFL induction were also demonstrated in arsenic‐treated cells with Petrosiol E pretreatment at 0.6, 1.25, and 2.5 µg mL^−1^, especially at 2.5 µg mL^−1^ (Figure 6F), in agreement with the data on neurite outgrowth determination (Figure 6D,E). Together, our results unveiled a novel role of Petrosiol E in combating oxidative stress and in promoting neuronal differentiation in arsenic‐treated PC12 cells through enhancing the cellular antioxidation capability.

## 3 Discussion

Neurite loss is a common cause of neuron injuries, which represents a target for the therapeutics of neuronal disorders.^14^ Due to a limited entry capacity to cross the BBB and a poor stability of nerutrophins, and their translational applications were remarkably restricted. In the meantime, other alternative approaches are accelerated for experimental and clinical studies including natural compounds.^32^, ^33^ Of the natural compounds, Petrosiol E was documented to induce neuron differentiation, albeit with unknown molecular mechanisms.^15^ Due to the limited quantity of Petrosiol E in nature, mechanistic and translational studies have been greatly reined for years. Until recently, we realized the artificial synthesis of Petrosiol E via the total synthesis, which opened the avenue to scrutinize the efficacy and mode of action of Petrosiol E in aiding neuron differentiation. To this end, in the current study, we endeavored to inspect the functions of Petrosiol E using PC12 cells, an established cell model for neuron‐like differentiation study.^34^ For the first time, we identified a dual role of Petrosiol E in inducing the differentiation of neuronal progenitors and in protecting them against oxidative stress as well. On one hand, Petrosiol E showed a robust ability to promote the neurite growth even at low concentrations. On the other hand, Petrosiol E also greatly protected cells from arsenic‐induced neurotoxicity, and Petrosiol E even enhanced neuronal differentiation under arsenic‐conducted toxicity.

To our knowledge, this is an innovative study by recognizing a novel compound Petrosiol E that reveals a pronounced capability to promote neuronal differentiation. Our data showed that Petrosiol E at low concentrations enhanced cell viability, whereas it favored cell differentiation with a concurrent decline of proliferation at higher concentrations (below 5 µg mL^−1^). It should be noted that the induction of differentiation and repression of cell division did not bring about cell death, highlighting the function of Petrosiol E in promoting neuronal differentiation. Nonetheless, slight cytotoxicity was observed in PC12 cells treated with Petrosiol E at the concentration ≥5 µg mL^−1^, pinpointing a reasonable dose range for Petrosiol E in promoting neuronal differentiation. Additionally, we compared Petrosiol E with other compounds that were previously reported to promote neuronal differentiation in PC12 cells.^21^, ^35^, ^36^ As shown in Figure S3 (Supporting Information), Petrosiol E even exerted a greater effect on NEFL induction than other 3 compounds, i.e., Ferulic acid, Berberine and Ginsenoside Rd, at the same concentration.

As the master regulator to drive a large battery of antioxidant genes, Nrf2‐mediated signaling pathways are crucial to combat oxidative stress.^37^ Meanwhile, mounting evidence suggests that Nrf2 is also involved in neuron differentiation.^38^, ^39^, ^40^ For example, primary neurons isolated from Nrf2‐deficient mice showed retarded neurite outgrowth, compared to those from wildtype mice.^26^ In contrast, forced expression of Nrf2 further potentiated SH‐SY5Y cell differentiation.^26^ To recognize the mechanisms responsible for Petrosiol E‐mediated dual role in neuronal progenitors, we here elaborated the signaling pathways linking the role of Nrf2 in inducing neuronal differentiation and in combating oxidative stress. In the current study, Nrf2 was first characterized to be activated by Erk1/2 and Akt phosphorylation in neuronal progenitor cells upon Petrosiol E treatment. In support of our findings, previous studies also documented the activation of Nrf2 downstream of Erk1/2 and PI3K‐Akt through various mechanisms, despite unclear regulation among them.^28^, ^41^, ^42^, ^43^ With the activation of Nrf2, neuronal differentiation was greatly induced, consistent with previous reports on the proneuron differentiation function of Nrf2.^35^, ^44^ At the same time, HO‐1, an Nrf2 downstream target of Nrf2, was induced by Petrosiol E to protect cells from oxidative stress. As a result, Petrosiol E harbored an ability to promote neuronal differentiation and simultaneously to combat oxidative stress through enhancing the cellular antioxidation responses, even under arsenic‐induced neurotoxicity. It should be noted that Petrosiol E may exert the antioxidant function through other mechanisms that need to be characterized in the future.

## 4 Conclusion

This study unearthed a vital function of Petrosiol E in inducing the differentiation of neuronal progenitors and in protecting them against arsenic‐induced oxidative stress in PC12 neuronal progenitor cells. With regard to the molecular mechanisms, Petrosiol E was uncovered to enhance the activity of Nrf2 in driving neuronal differentiation and in combating oxidative stress through Erk1/2 and Akt signaling. On one hand, enhanced Nrf2 activity would accelerate neuronal differentiation. On the other hand, enhanced Nrf2 activity would also facilitate the cellular antioxidation responses. The latter mechanism was verified in cells under arsenic‐induced neurotoxicity. It is worth noting that other mechanisms driven by Petrosiol E may also account for its dual role in neuronal differentiation and against oxidative stress. Additionally, Petrosiol E together with RA also displayed a capability to induce the differentiation of ES cells into neural ectoderm. With this pilot study, more efforts are needed to address Petrosiol E‐centered scientific and translational issues in the future.

## 5 Experimental Section

*Petrosiol E Synthesis*: Petrosiol E was synthesized from natural D‐xylose in ten steps using the carbohydrate chiral template approach, and the detailed procedures were described in the previous report.^16^ The compound purity used in this research met the elemental analysis requirement, i.e., 99.5% based on the high performance liquid chromatography (HPLC) detection. The chemical structure of Petrosiol E is depicted in Figure 1A. Petrosiol E was dissolved in dimethyl sulphoxide (DMSO) for further experiments.

*PC12 Cell Culture and Neuronal Differentiation*: The rat adrenal phenochromocytoma cell line PC12 was obtained from the Shanghai Cell Bank of Type Culture Collection of Chinese Academy of Science. Cells were cultured in RPMI 1640 medium (GIBCO Life Technologies, CA, USA), supplemented with 6% fetal bovine serum (FBS, GIBCO), 6% Horse serum (HS, GIBCO), and 100 U mL^−1^ penicillin‐streptomycin (GIBCO) at 37 °C under 5% CO_2_. For differentiation experiments, PC12 cells were cultured in differentiation medium: PRMI1640 medium with 1% HS, 1% FBS, and 100 U mL^−1^ penicillin‐streptomycin. Cells were first seeded on plates overnight and then replaced to differentiation medium for further experiments. Notably, the medium of 2.5 µg mL^−1^ Petrosiol E contained less than 0.1% DMSO. Medium containing 0.1% DMSO did not incur any toxicity to cells and change the status of cell differentiation, compared to the complete blank control. Thus, medium containing 0.1% DMSO here served as the vehicle control (designated as control or ctrl in the text and figures).

*Cell Viability Assay*: Cell viability was determined through the MTT assay (Solarbio, Beijing, China). Briefly, PC12 cells were seeded in 96‐well plates with 6 × 10^3^ per well overnight. Then, cells were cultured with Petrosiol E at various concentrations over the time course. Then, MTT was added for additional 4 h. Afterward, aspirated the medium and added 100 µL DMSO to each well. At the end, the absorbance of each sample was measured on a microplate reader (Varisosaka Flash).

*Cell Proliferation Determination*: BrdU assay was a direct indication of cell replication.^45^ BrdU was a derivative of thymine that could replace thymine during DNA synthesis for cell division, and incorporated BrdU could be recognized by its monoclonal Ab followed by immunochemical assessment.^45^ After cells were incubated with Petrosiol E at different concentrations and different time points, immunofluorescence was measured using a commercial assessment kit following the instructions provided by the manufacturers (Roche, IN, USA).

*ES Cell Culture and Differentiation Induction*: CCE was a mouse embryonic stem cell line,^46^ cultured in ES culture medium containing high glucose Dulbecco's modified Eagle's medium (DMEM) , 15% FBS, 1% nucleosides, 1% glutamine, 1% nonessential amino acids, 100 U mL^−1^ penicillin‐streptomycin, 1.0 × 10^3^ U mL^−1^ leukemia inhibitory factor, and 1.0 × 10^−4^ mol L^−1^ β‐mercaptoethanol at 37 °C with 5% CO_2_. Plates were precoated with 0.1% gelatin before use. For the differentiation into neural ectoderm, cells were allowed to form embryonic body (EB).^47^ After 4 d, 5 µmol L^−1^ RA was added to the culture medium with or without 2.5 µg mL^−1^ Petrosiol E. After additional 4 d, EBs were dispersed to single cells and cultured in N2 medium including DMEM‐F12, N2 supplement, 1% glutamine, and 100 U mL^−1^ penicillin‐streptomycin. On day 12, cells were collected for further analyses.

*Cell Death Assessment*: Cell death was evaluated by flow cytometry (FACS) analysis with PI and Annexin V staining (BD Biosciences, CA, USA). In brief, cells were seeded in 12‐well plates with 1 × 10^5^ cells per well. After different treatments, cells were collected and washed with phosphate buffer saline (PBS), followed by PI and Annexin V staining according to the instructions provided by the manufacturer. The stained cells were analyzed through fluorescence microscope (Axioscope A1, ZEISS) and subjected to FACS analysis on a BD FACSCalibur platform (BD) following the standard protocols, as previously described.^48^

*ROS Determination through Dichlorofluorescein Diacetate (DCFH‐DA)*: The endogenous ROS levels were assayed using the DCFH‐DA probe. Briefly, 6 × 10^3^ cells were cultured in 96‐well plates and DCFH‐DA (Sigma‐Aldrich, MO, USA) was added into each well at a final concentration at 10 × 10^−6^ mol L^−1^ for 30 min. Afterward, cells were washed three times with PBS and then treated with Petrosiol E at 0.6, 1.25, and 2.5 µg mL^−1^. Finally, DCF fluorescence was monitored using a microplate reader (Varisosaka Flash) at 1, 3, and 6 h. The excitation and emission wavelengths were 488 and 525 nm, respectively.

*Assessment of Neurite Bearing Cells*: According to the established method described previously,^49^ cells were defined as neurite bearing cells if they harbored at least one branch with the length greater than 5 nm. In this study, ≈100 cells were screened in three randomly picked up fields. Afterward, the percentage of neurite‐bearing cells was calculated by normalizing to the total number of cells in each group.

*Gene Knockdown of Nrf2 Expression*: Endogenous Nrf2 expression was knocked down through shRNA‐mediated gene silencing. Briefly, 8 × 10^5^ cells were seeded on 12‐well plates 12 h prior to plasmid transfection. Cells were transfected with 1.6 µg Nrf2‐selective shRNA‐expressing plasmids using Lipofectamine2000 (Invitrogen, CA, USA). Afterward, the medium was replaced 6 h post‐transfection, and the cells were cultured for additional 24 h. The sequence of Nrf2 shRNA#1 is CCGGGCTCGCATTGATCCGAGATATCTCGAGATATCTCGGATCAATGCG AGCTTTTTG, and the sequence of Nrf2 shRNA#2 is CCGGCCCGAATTACAGTGTCTTAATCTCGAGATTAAGACACTGTAAT TCGGGTTTTTG. Cells were also transfected with scrambled shRNA control plasmid. The efficacy of gene knockdown was examined with Western blotting.

*RNA Extraction and qRT‐PCR Analysis*: Total RNAs were extracted from cells using the Trizol reagent according to the instructions from the manufacturer (Invitrogen). The mRNA expression of interest genes were determined through qRT‐PCR analysis using SYBR Green qPCR master mix (Promega, WI, USA). The primers used in the study were as follows: Rat HO‐1 (forward, 5′‐TGCTCGCATGAACACTCTG‐3′; reverse, 5′‐TCCTCTGTCAGCAGTGCCT‐3′); Rat Gapdh (forward, 5′‐AACCTGCCAAGTATGATGAC‐3′; reverse, 5′‐GGAGTTGCTGTTGAAGTCA‐3′); mouse Dcx (forward, 5′‐CCATTGACGGATCCAGGAAG‐3′; reverse, 5′‐TCTGGCTTGAGCACTGTTGC‐3′); mouse Olig2 (forward, 5′‐ACAGACCGAGCCAACACCAG‐3′; reverse, 5′‐CGGGCAGAAAAAGATCATCG‐3′); mouse Sox1 (forward, 5′‐CCTCGGATCTCTGGTCAAGT‐3′; reverse, 5′‐GCAGGTACATGCTGATCATCTC‐3′); mouse Gapdh (forward, 5′‐AAGGTCATCCCAGAGCTG‐3′; reverse, 5′‐GCCATGAGGTCCACCACCCT‐3′). Gapdh was used as a loading control for the normalization of relative expression of interest genes.

*Western Blotting Analysis*: Cells after treatment were harvested and washed twice with PBS. Total proteins were extracted from cells with ice‐cold RIPA lysis buffer (Solarbio) containing protease inhibitor cocktail (Roche) and phosphatase inhibitor (Solarbio). Then, equal amounts of proteins were subjected to 8–12% SDS‐PAGE and Western blot analysis, as described previously.^50^ The Abs used in this study were listed below, anti‐GAP 43 Ab (1:500, Proteintech, Wuhan, China), anti‐NEFL Ab (1:1000, Proteintech), anti‐MAP2 Ab (1:1000; Proteintech), anti‐β‐actin Ab (1:5000, Proteintech), anti‐Nrf2 Ab (1:1000; Proteintech), anti‐H3 Ab (1:1000; Proteintech), anti‐HO‐1 Ab (1:1000; Proteintech), antimitogen‐activated protein kinase 1 (Erk1) Ab (1:1000; Proteintech), antiphosphorylated Erk1/2 Ab (1:500, Santa Cruz Biotechnology), antiserine/threonine kinase (Akt) Ab (1:1000; Proteintech), and antiphosphorylated Akt Ab (1:1000, Cell Signaling Technology).

*Immunofluorescence Staining*: The protein content of NEFL was assessed in PC12 cells using the technology of immunofluorescence staining, as previously described.^51^ Briefly, postcellular treatment, cells were fixed in 2% PBS‐buffered formaldehyde, followed by washing with PBS. Afterward, fixed cells were blocked with 5% FBS in PBS for 20 min and then incubated with an Ab against NEFL (1:100, proteintech, Wuhan, China). Immunocomplexes were visualized by FITC‐conjugated secondary Abs on a laser scanning confocal microscope (TCS SP5, Leica).

*SOD and Catalase Activity Assay*: Cells were inoculated in 12‐well plates and pretreated with Petrosiol E at 0.6, 1.25, and 2.5 µg mL^−1^ for 1 h, and were further treated with 1.3 µg mL^−1^ arsenic for additional 24 h. SOD activity was assessed using a SOD activity assay kit (Dojindo Lab, Tokyo, Japan), and catalase activity was determined by a catalase assay kit (Nanjing Jiancheng Bioengineering Institute, Nanjing, China), following the instructions from the manufacturers.

*Statistical Analysis*: SPSS statistics 17.0 software package was used to analyze the experimental data. The difference of the individual treated group relative to the untreated control was assessed using independent *t*‐test, and the significance of mean difference for two or more treatment groups relative to the untreated control was examined by one‐way ANOVA test. All experimental data were shown as mean ± standard deviation. In this study, *P* value less than 0.05 was considered statistically significant.

## Conflict of Interest

The authors declare no conflict of interest.

## Supporting information

Supplementary
