# Supplementary material for: An Important Function of Petrosiol E in Inducing the Differentiation of Neuronal Progenitors and in Protecting Them against Oxidative Stress
Source: Adv Sci (Weinh). 2017 Jul 5;4(10):1700089. doi: 10.1002/advs.201700089 (PMC5644239; doi:10.1002/advs.201700089)
Supplement: Supplementary file 1 — Supplementary [file ADVS-4-na-s001.pdf]

Copyright WILEY-VCH Verlag GmbH & Co. KGaA, 69469 Weinheim, Germany, 2017.

ADVANCED SCIENCE
Open Access

Supporting Information

for *Adv. Sci.,* DOI: 10.1002/advs.201700089

An Important Function of Petrosiol E in Inducing the Differentiation of Neuronal Progenitors and in Protecting Them against Oxidative Stress

Jing Liu, Linlin Wang, Yuguo Du, and Sijin Liu**

Supplementary Figures

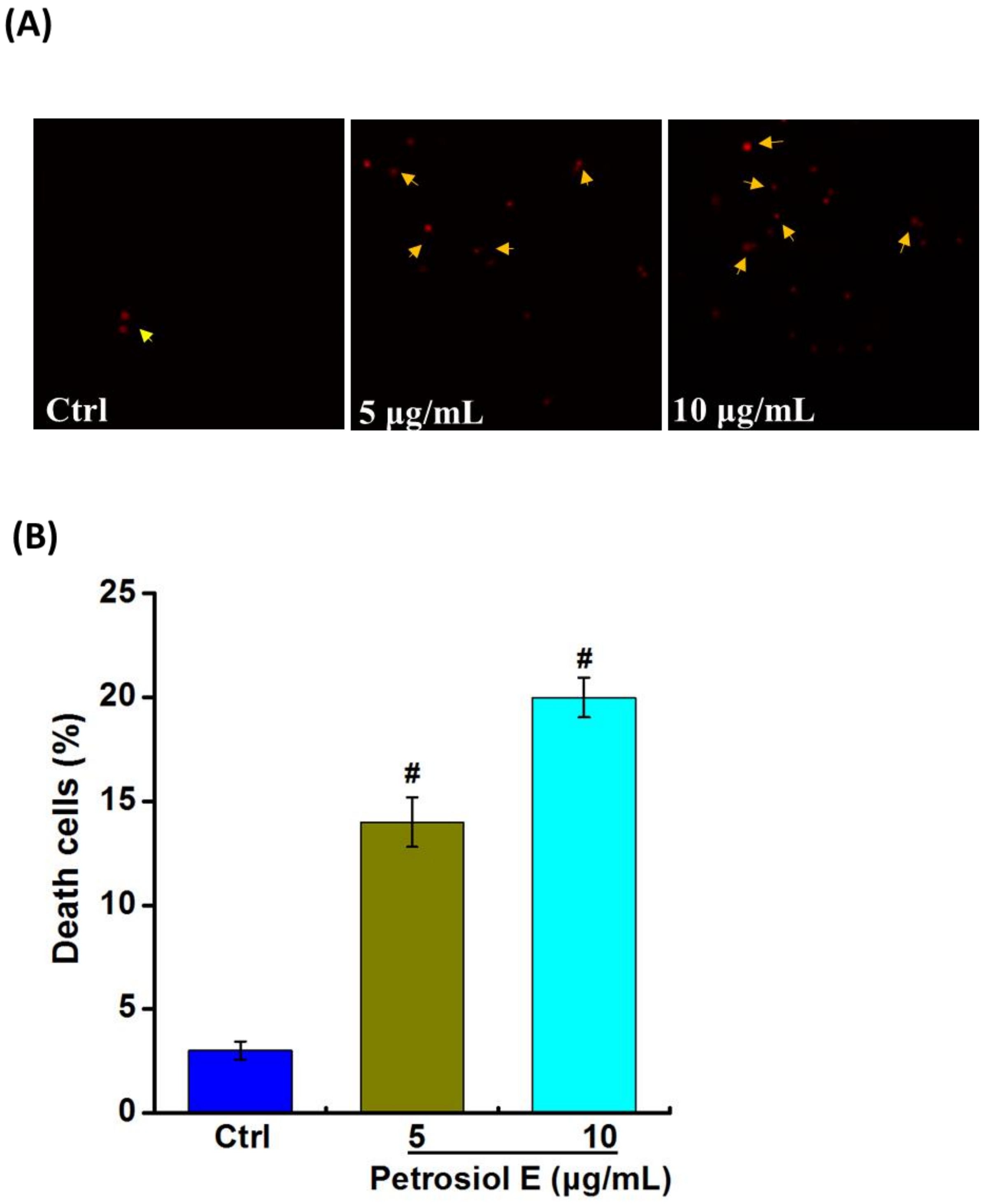

Supplementary Figure 1. Cell death assessment upon Petrosiol E. (A) PI staining of PC12 cells upon Petrosiol E treatment at 5 and 10 µg/mL for 24 h. (B) Quantitative analysis of dead cells after PI staining. *: P<0.05, relative to control. #: P<0.001, relative to control.

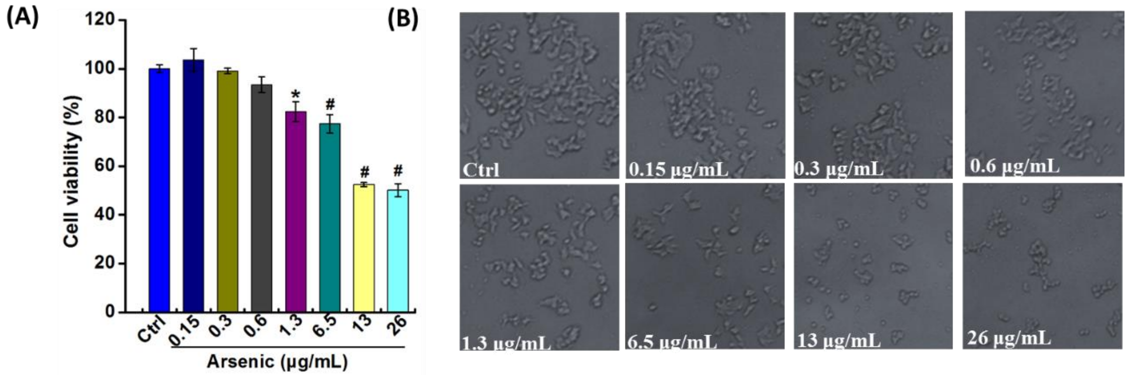

Supplementary Figure 2. Cytotoxicity of PC12 cells treated with arsenic at different concentrations. (A) Cell viability assessment of PC12 cells upon arsenic treatment for 24 h through the MTT assay. (B) The morphology of PC12 cells after arsenic exposure for 24 h. *: $P<0.05$; #: $P<0.001$, relative to control.

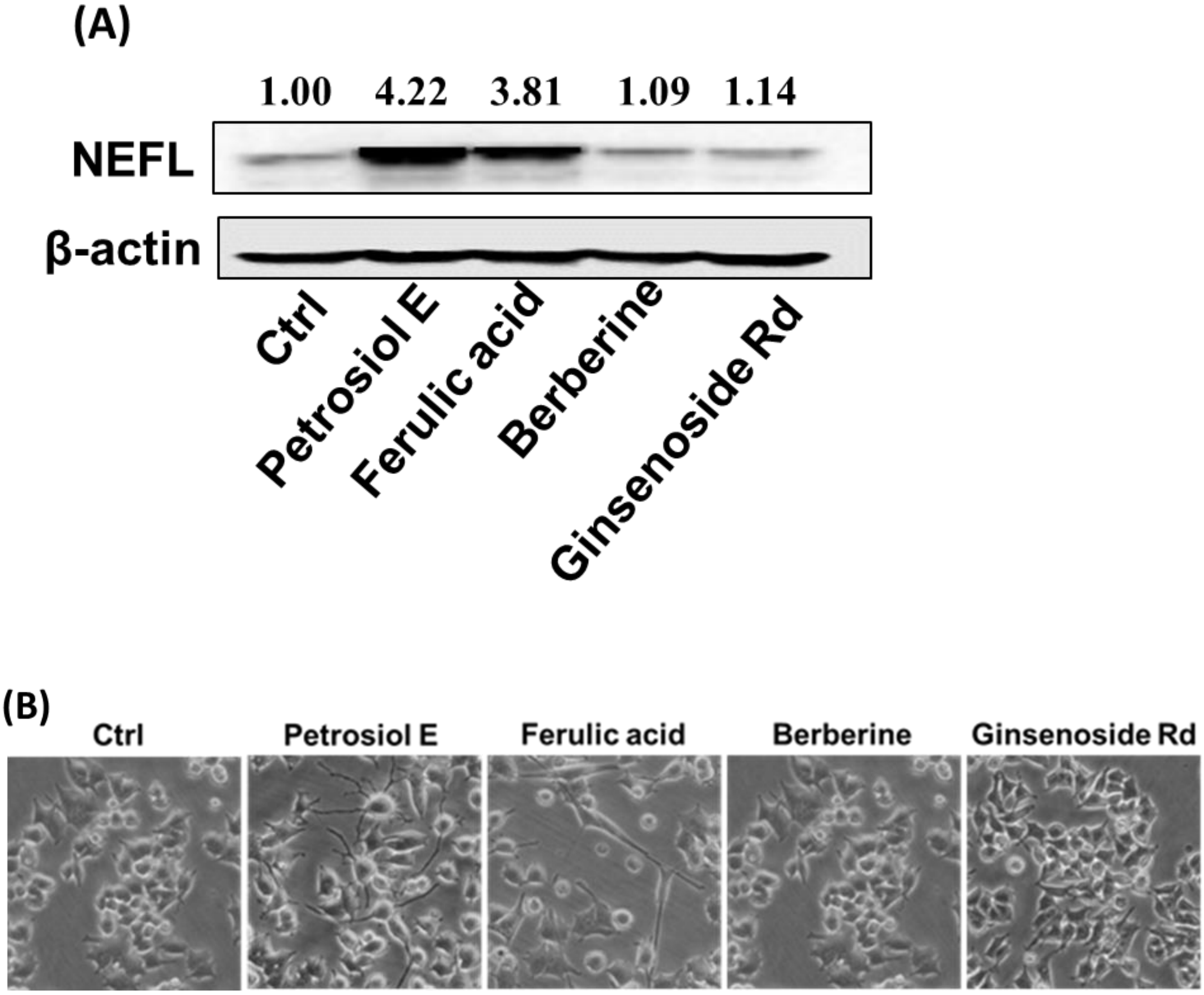

Supplementary Figure 3. Neurite outgrowth of PC12 cells upon different compounds. (A) NEFL concentrations and (B) morphological alterations in PC12 cells treated with Petrosiol E, Ferulic acid, Berberine and Ginsenoside Rd at 2.5 µg/mL for 3 days.
